# Supplementary material for: Nutritional Considerations of Irish Performance Dietitians and Nutritionists in Concussion Injury Management
Source: Nutrients. 2024 Feb 9;16(4):497. doi: 10.3390/nu16040497 (PMC10891776; doi:10.3390/nu16040497)
Supplement: Supplementary file 1 [file nutrients-16-00497-s001.zip › S2 - Table S1 1123.pdf]

Table S1. Thematic Analysis (Phase 2). Initial code generation based on theoretical assumptions and research aims (semantic approach).

Code	Code Description
**CR: Concussion Injury Report**	
CR 1	Injury mentioned.
CR 2	Interviewer probed (was not mentioned as a common injury)
CR 3	Athlete
**CA: Concussion Awareness**	
CA1	Head injury/ concussion event awareness/ communication
CA2	PN dealings with concussion injuries in athlete
CA3	Blasé attitude to concussion injuries in rugby
CA4	Timeframe for PN to support athletes' (acute period (<14 days))
CA5	PN previously experienced a concussion as an athlete
CA6	PN no mention until probed (did not mentioned as a common injury)
CA7	Symptoms/ recovery need (rest, nausea…)
CA8	Protocols increasing injury risk/ sport – hydration/ drying out
**NC: Nutrition Consideration - Concussion Support**	
NC1	PN poor awareness/ no consideration/ no mention/ see link
NC2	PN awareness – mentions/ logically considers nutrition specific to concussion/ researching
NC3	PN awareness – mentions referring out for nutrition support
NC4	PN new awareness/ comfort/ reassurance/ increased buy-in – nutrition
NC5	PN's Concussion Nutrition Protocols
**NP: Nutrition Protocol - Concussion Support**	
NP1	Poor knowledge on concussion nutrition protocols
NP2	Implementation of strategic overall nutrition support protocol
NP3	Implementation of dietary protocols to support brain healing/ recovery
NP4	Implementation novel protocols that may support brain healing/ recovery
NP5	No specific nutrition strategy for concussion recovery
NP6	Appetite – not an issue in concussion
NP7	Athlete adherence concussion protocols – reality/ evidence markers
NP A.	A. Nutrition protocols that may support concussion recovery
**NP5 - C**	**Creatine supplementation protocols**
C1	No true awareness/ knowledge
C2	Awareness of potential creatine role in concussion recovery
C3	Application is not a strategic concussion recovery support protocol?
C4	Protocol specific to increase intake of creatine post-concussion event
**NP5- FO**	**Fish Oil - Omega 3 supplementation**
FO1	No true awareness/ knowledge – application purpose
FO2	Awareness of omega-3 fish oils potential role in concussion
FO3	Application is not a strategic concussion recovery support protocol?
FO4	Protocols to increase omega 3 intakes post-concussion event

**NP5 -P**	**Probiotic - supplementation**
P1	No true awareness/ knowledge – application purpose
P2	Gut-brain support - Anecdotal evidence
**NP5 - Cr**	**Carb reloading protocol**
C-r1	Carbohydrate reloading – PN purpose uncertainty
C-r2	Protocol to carb-reload post-concussion event

Abbreviation notes: ***CR*** **=** *Concussion report,* ***CA*** **=** *Concussion awareness,* ***NC*** **=** *Nutrition Consideration (concussion support),* ***NP*** **=** *Nutrition Protocols (concussion support),* ***C*** **=** *Creatine supplementation,* ***FO*** **=** *Fish Oil - Omega 3 supplementation,* ***P*** **=** *Probiotic – supplementation,* ***C-r*** **=** *Carb reloading.*
