# Supplementary figures and images for: Nutritional Considerations of Irish Performance Dietitians and Nutritionists in Concussion Injury Management
Source: Nutrients. 2024 Feb 9;16(4):497. doi: 10.3390/nu16040497 (PMC10891776; doi:10.3390/nu16040497)

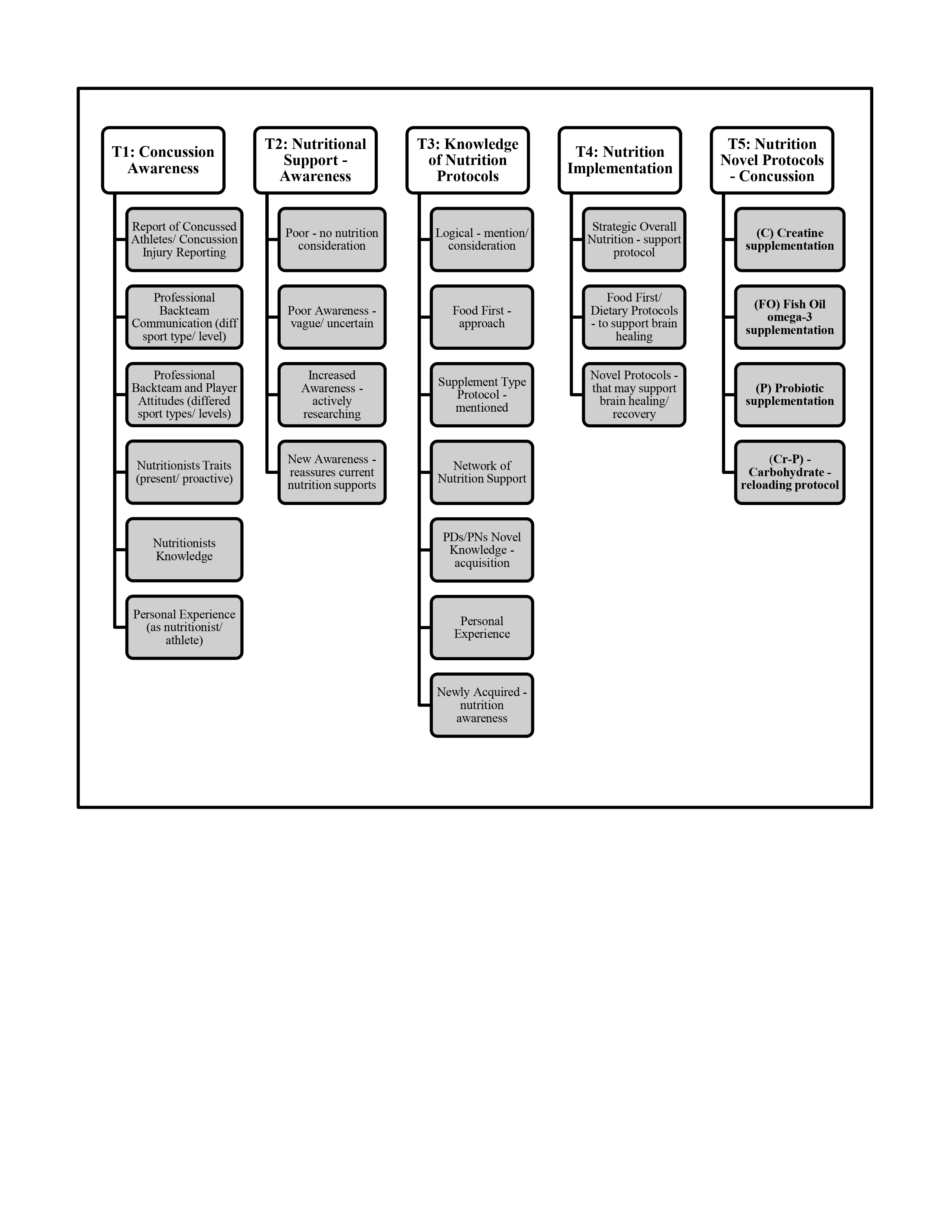

Supplement: Supplementary file 1 [file nutrients-16-00497-s001.zip › Supp 1 Figure 1 (A) P3 Thematic analysis 23.jpg]

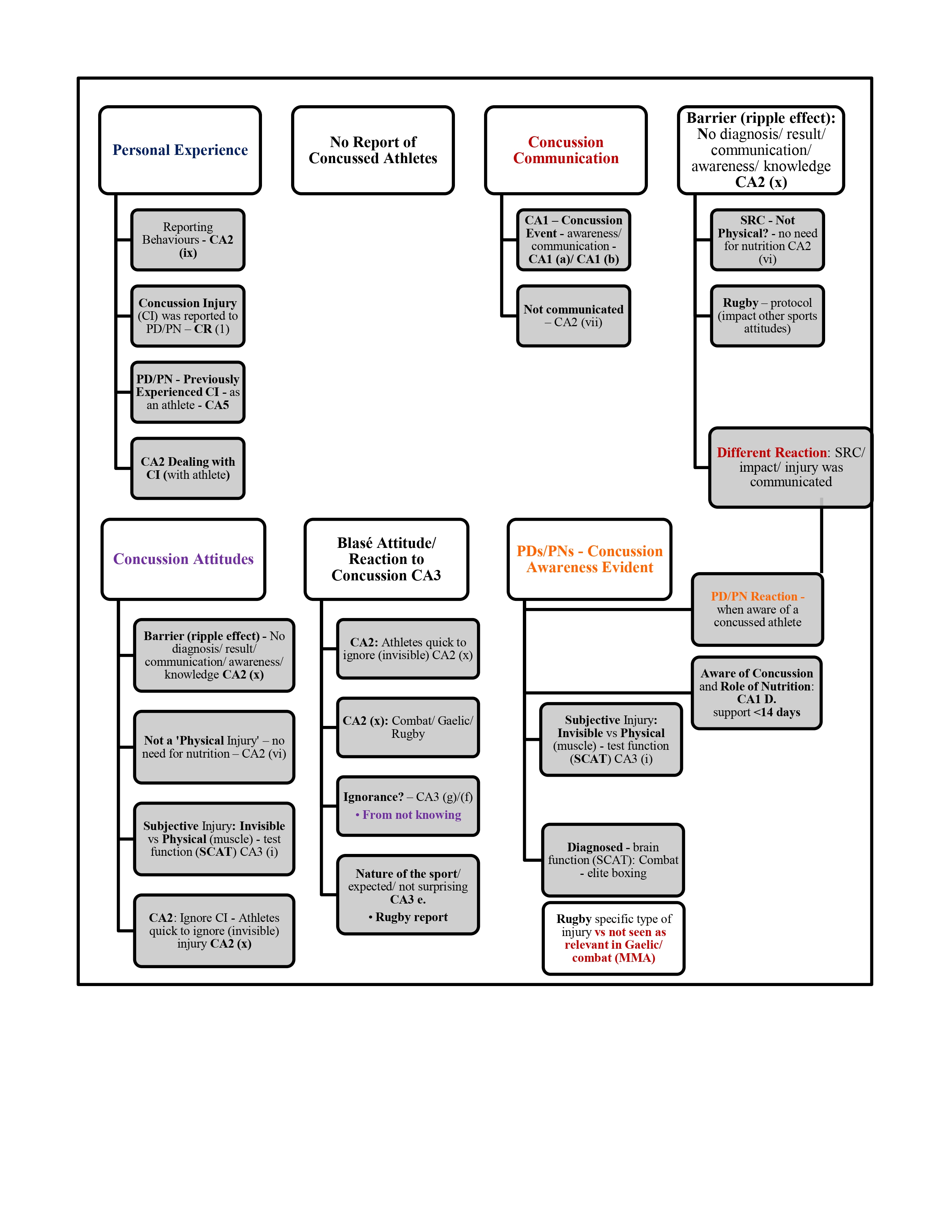

Supplement: Supplementary file 1 [file nutrients-16-00497-s001.zip › Supp 1 Figure 1 (B) P4 Thematic analysis 23.jpg]

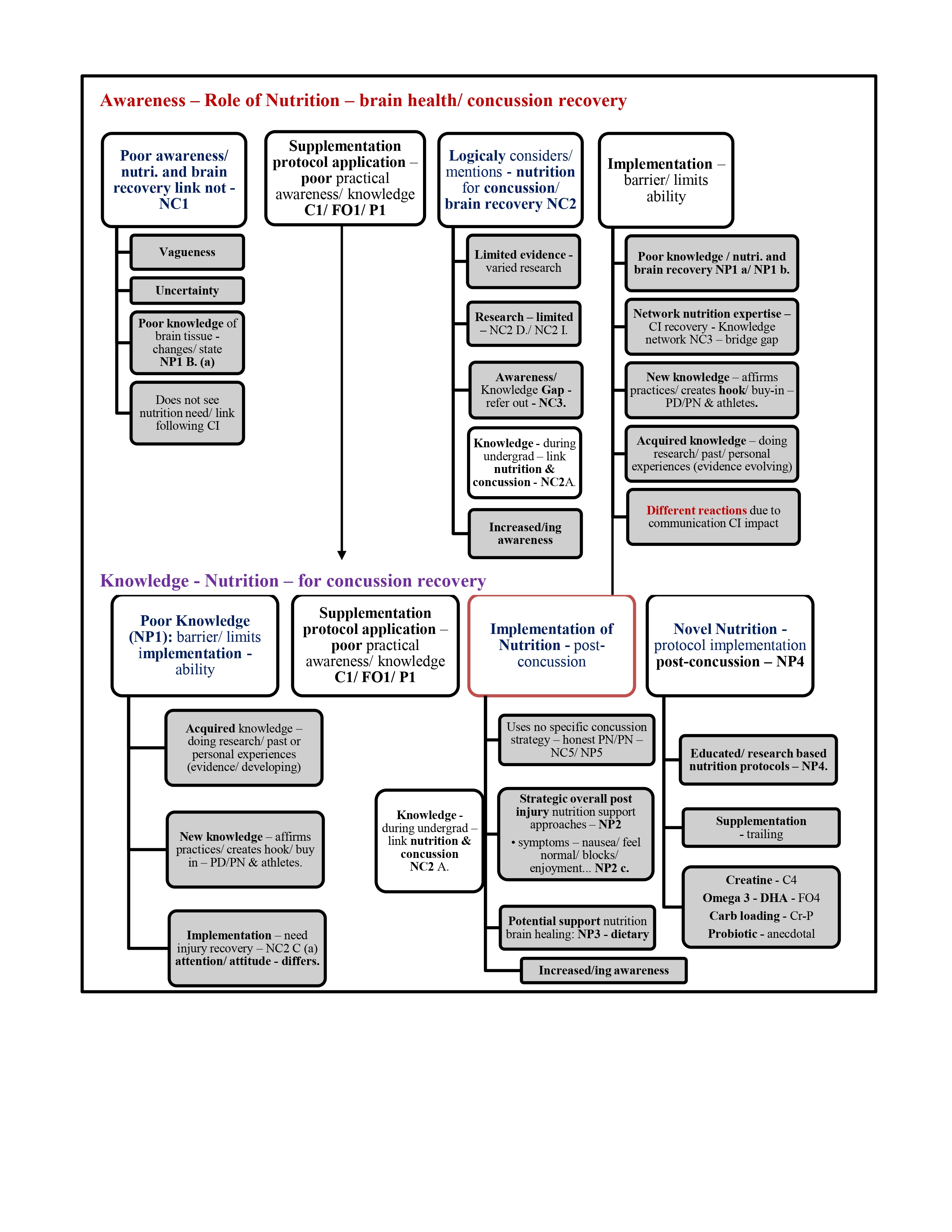

Supplement: Supplementary file 1 [file nutrients-16-00497-s001.zip › Supp 1 Figure 1 (C) P4 Thematic analysis 23.jpg]
